# Relative Age Effects in Male Japanese Professional Athletes: a 25-Year Historical Analysis

**DOI:** 10.1186/s40798-020-00277-4

**Published:** 2020-10-06

**Authors:** Nao Sasano, Yoichi Katsumata, Hiroki Nakata

**Affiliations:** 1 Faculty of Human Life and Environment, Nara Women’s University, Kitauoya-Nishimachi, Nara, 630-8506 Japan; 2 Faculty of Applied Bio-Science, Tokyo University of Agriculture, Tokyo, Japan

**Keywords:** RAE, Sport, Japan, Adolescent

## Abstract

**Background:**

The mechanisms underlying the relative age effect (RAE), a biased distribution of birth dates, in sport events have been investigated for more than two decades. The present study comprised an historical analysis involving the most recent quarter-century (1993–2018) on RAEs among Japanese male professional athletes (soccer, baseball, basketball, and volleyball) to clarify how the RAEs changed over time.

**Methods:**

Birth data were obtained from 7805 Japanese male professional athletes registered in 1993, 2001, 2010, and 2018. The athletes were divided into four groups based on their month of birth: quartiles Q1 (April–June), Q2 (July–September), Q3 (October–December), and Q4 (January–March of the following year). In addition, based on the data in 1993 for soccer and baseball and in 2010 for basketball and volleyball, the expected numbers of players were calculated in 2001, 2010, and 2018 for soccer and baseball, and 2018 for basketball and volleyball.

**Results:**

Significant RAEs were observed among soccer and baseball players in 1993, 2001, 2010, and 2018, and strong tendencies of RAEs were found among basketball and volleyball players in 2010 and 2018. The magnitudes of the RAEs in soccer, baseball, and volleyball decreased over time, but not in basketball.

**Conclusion:**

The exact reasons for the decreasing or unchanging RAEs among these professional players remain unclear, but socio-cultural factors, such as low birthrates and the popularity of sports in Japan, might be related to the changing RAEs.

## Key Points

- Significant RAEs were observed among Japanese professional soccer and baseball players registered in 1993, 2001, 2010, and 2018.
- The magnitude of RAEs in soccer, baseball, and volleyball decreased over time.
- Socio-cultural factors, such as low birthrates and the popularity of sports in Japan, might be related to the changing RAEs.

## Background

For over a quarter of century, the “relative age effect” (RAE) has been investigated as one of the factors influencing sporting success. Relatively older children in a particular age group are more likely to achieve sporting success, compared with relatively younger children. Within the same age category, there can be a difference of almost a full year between the oldest and youngest children. RAEs have been confirmed in many sports, including soccer [1, 2], baseball [3, 4], basketball [5, 6], handball [7], swimming [8, 9], track and field [10, 11], sumo wrestling [11], rugby [12], and alpine ski racing [13, 14]. The attributes of greater height, mass, aerobic power, muscular strength, endurance, and speed provide performance advantages in most sports, giving relatively older children advantages in sporting ability, psychological confidence, instruction, and playing time [15]. To date, a variety of sports contexts differing in age categories and cultures have been assessed to examine RAEs (see a meta-analytical review, [15]).

In addition to physiological and psychological factors, the competition principle has also been considered as an important factor affecting RAEs. According to Musch and Grondin [16], “Competition will come from the number of players available for the places, and this number will depend on the popularity of a given sport in a given country (p. 154).” Thus, the level of competition is associated with the popularity of the sport. For example, in Japan, soccer, baseball, basketball, and volleyball are very popular among male elementary and junior high school students [17]; however, handball, rugby, badminton, and American football are not so popular. Indeed, Nakata and Sakamoto [11] showed significant RAEs among Japanese players in soccer, baseball, basketball, and volleyball, which were major sports in Japan, but no significant RAEs were observed in handball, rugby, badminton, American football, or golf. Consequently, if a sport does not need a physical advantage and is not so popular in a given country, RAEs may not be observed. In addition, it takes several years or decades for a sport to gain popularity in a given country. Therefore, historical analysis is needed to know the beginning of RAEs in a country and compare differences in the magnitude of RAEs among generations, and to be considered based on socio-cultural factors. To date, there have been several studies examining RAEs from a historical perspective [18–22]. For example, Nakata and Sakamoto [22] investigated the existence of RAEs in Japanese professional baseball players born in 1911–1980. They reported that significant RAEs were observed among Japanese professional baseball players born in the 1910s and onward, and the magnitude of RAEs increased with time. These studies suggest that the magnitude of RAEs changed with time and that socio-cultural factors, such as international competitions, and media coverage may have markedly contributed to this.

However, after a thorough literature search, we do not know of any study conducting historical analysis of recent generations of RAEs in a given country, rather than the beginning of RAEs [18–22]. Moreover, previous historical studies only examined RAEs on one sporting event. We consider that RAEs on some sports should be evaluated simultaneously in a given country to clarify the socio-cultural factors relating to RAEs. In fact, demographics have clearly changed compared with 50 years ago. For example, in Japan, the number of children is markedly decreasing (Ministry of Internal Affairs and Communications, the Statistics Bureau and the Director-General for Policy Planning of Japan [23]), and recent RAEs on some sports might change over time. Based on this research background, the objective of the present study was to investigate the characteristics of RAEs over a recent quarter-century (1993–2018) among professional soccer, baseball, basketball, and volleyball players simultaneously in Japan. Japan has applied a unique annual-age grouping for education since 1886, basing group assignment on student birthdates between April 1 (the “new” year) and March 31 of the following year among elementary, junior high, senior high, and university (college) students and in government and company employment. Sports calendars also follow this system, giving Japanese children and adolescents born between April and June a relative age advantage over those born between January and March. We investigated whether the magnitude of RAEs in Japanese professional soccer, baseball, basketball, and volleyball, which are major male-dominated sports in Japan, changed over time.

## Method

### Samples

As mentioned above, soccer, baseball, basketball, and volleyball are very popular among male Japanese elementary and junior high school students [17]. Therefore, the present study focused on the RAEs in these professional sports. Data on professional Japanese soccer players (*N* = 3490 males) who played in the Japan Professional Football League (J-league) registered in 1993, 2001, 2010, and 2018 were extracted from official publications [24–27]. Data on professional Japanese baseball players (*N* = 3096 males) who played in Nippon Professional Baseball (NPB) registered in 1993, 2001, 2010, and 2018 were extracted from official publications [28–30]. Data on professional Japanese basketball players (*N* = 586 males) who played in the Japan Professional Basketball League (B-league) registered in 2010 and 2018 were extracted from an official publication [31] and a previous study [11]. Data on professional Japanese volleyball players (*N* = 633 males) who played in the Japan Volleyball League (V-league) registered in 2010 and 2018 were extracted from an official website [32] and a previous study [11]. Each league is the highest level in Japan.

### Data Analysis

Professional players were divided into four groups based on their month of birth: Q1 (April–June), Q2 (July–September), Q3 (October–December), and Q4 (January–March of the following year). Foreign players in the Japanese professional leagues were excluded because they had not passed through the Japanese school system. Chi-squared tests were applied to each group according to the four quarters to assess the significance of deviations from the expected number of births in each quarter. In line with previous studies [22, 33, 34], the expected distribution was calculated based on the general population of males in national birth statistics from 1962 to 1999 in Japan [23]. According to a previous study [11], the age range for calculating the general population was set at 12 years. For example, the age of players registered in 1993 was considered to be 18 to 30 years old, and we calculated the general population born between 1962 (i.e., 30 years old) and 1974 (i.e., 18 years old) (Supplementary Table 1). The effect size of chi-squared tests was also calculated in each group. To measure the effect size, Phi (φ) was defined as:

$$ \upvarphi \sqrt{\frac{\chi^2}{n}} $$

where *n* = the number of observations.

The odds ratio (OR) and 95% confidence intervals were then calculated to provide additional information for both quartile and half-year distributions, following a previous review article [15]. The ORs for the Q1, Q2, and Q3 vs. Q4 comparisons were interpreted as follows: OR < 1.22, 1.22 ≤ OR < 1.86, 1.86 ≤ OR < 3.00, and OR ≥ 3.00, indicating negligible, small, medium, and large effects, respectively [3, 35], which was recommended by sample size calculation [36]. In addition, based on the data in 1993 for soccer and baseball and in 2010 for basketball and 2018, the expected numbers of players were calculated for 2001, 2010, and 2018 in soccer and baseball, and for 2018 in basketball and volleyball. In this analysis, chi-squared tests were applied to the data for 2001, 2010, and 2018 in order to assess the changing of RAEs with time.

In the present study, we focused on only male players, because the number of female players was small. For example, there are only 4 female professional baseball teams in Japan, and the professional league only started in 2010 [37]. No female professional basketball league exists in Japan.

Statistical tests were performed using computer software (SPSS for windows ver. 22.0, SPSS). Significance was set at *p* < 0.05, and the *p* values are shown as *p* < 0.05, *p* < 0.01, and *p* < 0.001.

## Results

Table 1 presents the results of chi-squared tests for each sport and year. Significant RAEs of soccer and baseball were observed in 1993, 2001, 2010, and 2018. The effect size (Phi) in both sports gradually decreased over time. No significant RAEs of basketball and volleyball were observed at any ages, but the percentage of Q1 was the highest, and that of Q4 was the lowest. The effect size in basketball increased slightly over time, while that in volleyball decreased over time.

**Table 1:** Distribution of all professional players divided into quartiles in each sport

	Year	Q1	Q2	Q3	Q4	Total	*χ*^2^	Phi
Soccer	1993	129 (36.5%)	108 (30.6%)	57 (16.1%)	59 (16.7%)	353	47.17*	0.366
	[Predicted]	86	89	86	92			
	2001	277 (35.9%)	233 (30.0%)	153 (19.8%)	109 (14.1%)	772	79.74*	0.321
	[Predicted]	194	203	188	188			
	2010	331 (34.7%)	273 (28.6%)	212 (22.2%)	139 (14.6%)	955	78.46*	0.287
	[Predicted]	237	251	236	231			
	2018	467 (33.1%)	422 (29.9%)	294 (20.9%)	227 (16.1%)	1410	88.55*	0.251
	[Predicted]	354	369	350	337			
Baseball	1993	279 (34.7%)	262 (32.6%)	142 (17.7%)	120 (14.9%)	803	105.02*	0.362
	[Predicted]	196	203	195	210			
	2001	264 (36.1%)	224 (30.6%)	141 (19.3%)	102 (14.0%)	731	80.61*	0.332
	[Predicted]	183	192	178	178			
	2010	240 (32.8%)	212 (29.0%)	175 (23.9%)	104 (14.2%)	731	51.26*	0.265
	[Predicted]	181	192	181	177			
	2018	258 (31.0%)	236 (28.4%)	181 (21.8%)	156 (18.8%)	831	25.44*	0.175
	[Predicted]	209	218	206	199			
Basketball	2010	48 (30.4%)	44 (27.8%)	35 (22.2%)	31 (19.6%)	158	3.91	0.157
	[Predicted]	39	42	39	38			
	2018	137 (32.0%)	124 (29.0%)	84 (19.6%)	83 (19.4%)	428	17.65	0.203
	[Predicted]	107	112	106	102			
Volleyball	2010	39 (29.3%)	43 (32.3%)	28 (21.1%)	23 (17.3%)	133	6.27	0.217
	[Predicted]	33	35	33	32			
	2018	141 (28.2%)	123 (24.6%)	130 (26.0%)	106 (21.2%)	500	3.73	0.086
	[Predicted]	126	123	124	120			

The number in the second row is the predicted number of players obtained using the chi-squared test. *n* number of players, *χ*^*2*^ chi-squared value

^*^p < 0.001

Table 2 demonstrates OR and 95% confidence intervals for each group. The middle effects of OR were observed in Q1 and Q2 vs. Q4 for soccer in 1993, 2001, 2010, and 2018. The middle effects of ORs were observed in Q1 and Q2 vs. Q4 for baseball in 1993, 2001, and 2010, but the small effects of ORs were observed in Q1 and Q2 vs. Q4 for baseball in 2018. The small effects of OR were observed in Q1 and Q2 vs. Q4 for basketball in 2010 and 2018. The small effects of ORs were observed in Q1 vs. Q4 for volleyball in 2010 and 2018, and Q2 vs. Q4 for volleyball in 2010. In the data on half-year distributions (i.e., 1st vs. 2nd semesters), ORs gradually decreased with time in soccer, baseball, and volleyball, but not in basketball.

**Table 2:** Odds ratio and 95% confidence interval for each group

	Year	Q1 vs. Q4	Q2 vs. Q4	Q3 vs. Q4	1st vs. 2nd
Soccer	1993	2.19 (1.93–2.48)	1.83 (1.61–2.07)	0.97 (0.86–1.10)	2.04 (1.80–2.31)
	2001	2.54 (2.24–2.88)	2.14 (1.89–2.43)	1.40 (1.23–1.59)	1.95 (1.72–2.21)
	2010	2.38 (2.10–2.70)	1.96 (1.73–2.22)	1.53 (1.35–1.73)	1.72 (1.52–1.95)
	2018	2.06 (1.82–2.34)	1.86 (1.64–2.11)	1.30 (1.15–1.47)	1.71 (1.51–1.94)
Baseball	1993	2.33 (2.06–2.64)	2.18 (1.92–2.47)	1.18 (1.04–1.34)	2.06 (1.82–2.34)
	2001	2.59 (2.28–2.94)	2.20 (1.94–2.49)	1.39 (1.23–1.58)	2.01 (1.77–2.28)
	2010	2.31 (2.04–2.62)	2.04 (1.80–2.31)	1.68 (1.48–1.90)	1.62 (1.43–1.84)
	2018	1.65 (1.46–1.87)	1.51 (1.33–1.71)	1.16 (1.02–1.32)	1.47 (1.30–1.67)
Basketball	2010	1.55 (1.37–1.76)	1.41 (1.24–1.60)	1.13 (1.00–1.28)	1.39 (1.23–1.58)
	2018	1.65 (1.46–1.87)	1.49 (1.31–1.69)	1.01 (0.89–1.14)	1.56 (1.38–1.77)
Volleyball	2010	1.70 (1.50–1.93)	1.87 (1.65–2.12)	1.22 (1.08–1.38)	1.6 (1.41–1.81)
	2018	1.33 (1.17–1.51)	1.16 (1.02–1.32)	1.23 (1.08–1.39)	1.12 (0.99–1.27)

*1st* the first semester (Q1 and Q2), *2nd* the second semester (Q3 and Q4)

Table 3 shows the expected numbers of players in 2001, 2010, and 2018 for soccer and baseball, and in 2018 for basketball and volleyball, based on the data in 1993 for soccer and baseball and in 2010 for basketball and 2018. The results of chi-squared tests showed significant RAEs for soccer in 2001, 2010, and 2018; baseball in 2010 and 2018; and volleyball in 2018. These data indicate that the magnitudes of RAEs decreased over time.

**Table 3:** Number of players divided into quartiles in each sport

	Year	Q1	Q2	Q3	Q4	Total	*χ*^2^	Phi
Soccer	1993	129 (36.5%)	108 (30.6%)	57 (16.1%)	59 (16.7%)	353		
	2001	277	233	153	109	772	9.50*	0.111
	[Expected]	282	236	125	129	772		
	2010	331	273	212	139	955	25.64**	0.164
	[Expected]	349	292	155	159	955		
	2018	467	422	294	227	1410	24.11**	0.131
	[Expected]	515	431	228	236	1410		
Baseball	1993	279 (34.7%)	262 (32.6%)	142 (17.7%)	120 (14.9%)	803		
	2001	264	224	141	102	731	2.92	0.063
	[Expected]	254	238	129	110	731		
	2010	240	212	175	104	731	20.34**	0.167
	[Expected]	254	238	129	110	731		
	2018	258	236	181	156	831	23.20**	0.167
	[Expected]	288	271	147	125	831		
Basketball	2010	48 (30.4%)	44 (27.8%)	35 (22.2%)	31 (19.6%)	158		
	2018	137	124	84	83	428	1.87	0.066
	[Expected]	130	119	95	84	428		
Volleyball	2010	39 (29.3%)	43 (32.3%)	28 (21.1%)	23 (17.3%)			
	2018	141	123	130	106	500	20.24**	0.201
	[Expected]	147	162	105	86	500		

The number in the second row shows the expected number of players calculated from the number of players in 1993 for soccer and baseball, and in 2010 for basketball and volleyball. *χ*^*2*^ chi-squared value

^*^p < 0.05

^**^p < 0.001

## Discussion

In the present study, an historical analysis involving the most recent quarter-century (1993–2018) on RAEs among Japanese professional athletes was performed to clarify how the RAEs changed over time.

Relatively older players (i.e., Q1 players) may have greater opportunities for selection and experience in childhood because they are naturally heavier, taller, stronger, and faster; have greater endurance; and are more coordinated than relatively younger players during childhood [15, 16, 38], all of which translate into performance advantages in most sports. This has been often described as the “maturation-selection hypothesis” (see a review, [15]). Judging from Table 1, our data in four professional sports including soccer, baseball, basketball, and volleyball support these previous findings. That is, the percentage of Q1 among the four sports was the highest for 25 years, and that of Q4 was the lowest, although the effect size (Phi) gradually decreased over time. This was also supported by the OR data in Table 2. These data indicate that RAEs in fours sports existed for 25 years. Since the RAEs are influenced by player selection and training methods, it is necessary to promote the awareness of “adults (guardians, coaches, associations, etc.).” In other words, if adults have little knowledge about RAEs, relatively older players will be given priority and talented relatively younger players may be overlooked. Some previous studies reported cases to reduce RAEs [9, 39, 40]. For example, Mann and van Ginneken [40] reported a significant selection bias for the scouts of youth soccer in a no-age information group, and that bias remained when scouts knew the players’ dates of birth. The selection bias was eliminated when scouts watched the games knowing the shirt numbers corresponded to the relative ages of the players. However, with regard to the decrease in RAEs among professional players in Japan, it is unlikely that efforts to reduce the RAEs were made nationwide. Therefore, other factors need to be considered.

One of the possibilities is that the popularity of sports has decreased annually. Table 4 shows changes in the number of youth baseball teams, players, and players per team. Data on baseball were from official websites of the Nippon Junior High School Physical Culture Association [41] and Japan High School Baseball Foundation [42]. Since data from 1993 were not available, we used data from 1994. As a result, the number of youth players per team at junior high school clearly decreased in 2018. The numbers of teams at high school (hardball and rubber ball) were almost the same for 25 years. Therefore, we considered that the decrease in the OR for professional baseball players was partly related to the decrease in the popularity of baseball, especially in junior players. In contrast, youth baseball players who played at junior high school might tend to continue to play at high school. Katsumata et al. [3], focusing on Japanese youth baseball players in recreational levels at elementary school, junior high school, and high school, showed that significant RAEs were noted in recreational junior high school and high school players, but not in elementary school players, and the effect size became larger with increasing grade (0.063 in elementary school players, 0.151 in junior high school players, and 0.227 in high school players). Their data suggest that relatively younger players may gradually drop out from playing baseball even as recreational players. Delorme et al. [5] reported that the relative age was associated with sport dropout. They showed that relatively younger players in categories of 9–10 years old, 11–12 years old, and 13–14 years old tended to drop out from basketball, compared with relatively older players. Indeed, the present study did not directly elucidate the relationships between these backgrounds and effect sizes in professional baseball players; further studies are necessary to clarify the detailed mechanisms.

**Table 4:** Changes in the number of youth baseball teams, players, and players per team

	Year	Team (*n*)	Player (*n*)	Number of players per team (*n*)
Junior high school (rubber ball)	1994	8702	–	–
	2001	8391	321,692	38.3
	2010	8919	291,015	32.6
	2018	8384	166,800	19.9
High school (hardball)	1994	4104	142,481	34.7
	2001	4208	149,622	35.6
	2010	4115	168,488	40.9
	2018	3971	153,184	38.6
High school (rubber ball)	1994	627	14,373	22.9
	2001	549	12,892	23.5
	2010	482	11,014	22.9
	2018	428	8755	20.5

Next, we have to consider why the effect size of RAEs among soccer, baseball, and volleyball players decreased over time. As mentioned above, it is unlikely that efforts to reduce the RAEs were made nationwide. Therefore, other factors need to be considered. We proposed the acceleration of the age of peak height velocity (APHV) over time. Hermanussen et al. [43] reported that Japanese APHVs from 1948 to 2003 accelerated from 14.07 to 12.03 years old for boys and from 11.80 to 9.92 years old for girls. Yokoya and Higuchi [44] also reported that the Japanese APHV between 2006 and 2013 was 11.79 years old for boys and 9.55 years old for girls. Since these data cover up to 2013, the latest values of APHA should show even more acceleration. We hypothesized that if biological maturation through secondary sexual development is accelerated year by year, the timing for reducing RAEs might be more accelerated. In other words, the differences in physical performance, including aerobic power, muscular strength, endurance, and speed, between relatively older and younger children might be reduced or masked in adolescents. Indeed, no significant RAEs in height were observed among baseball players in recreational high school [3]. In future studies, it will be necessary to examine the relationship between the acceleration of APHV and RAEs. On the other hand, the magnitude of the RAEs only among basketball players remained unchanged over time. The reason remains unclear, but we also considered that the popularity of basketball is associated with the unchanged RAEs. The Japan Professional Basketball League (B-league) only started in 2016, although there were semi-professional basketball leagues in Japan before 2016. The start of the new professional league may have affected RAEs of basketball players. The issue of RAEs in basketball players should be resolved in the future, and it would be needed to examine whether RAEs in basketball change with time. If basketball becomes more popular, RAEs might be observed in the future. To prevent this, it is necessary to promote the awareness and understanding of adults (guardians, coaches, associations, etc.) for RAEs involved in young basketball players.

As a limitation of the present study, our finding might be specific to Japan, because many activities, sports-related or academic, are based on a unique cutoff date (April 1), which is not the case in other countries. Data on RAEs with an historical analysis should be examined in other countries.

## Conclusion

The present study comprised an historical analysis to clarify the characteristics of RAEs among Japanese professional soccer, baseball, basketball, and volleyball players over time. Significant RAEs were observed among soccer and baseball players in 1993, 2001, 2010, and 2018, and strong tendencies of RAEs were found among basketball and volleyball players in 2010 and 2018. In addition, the magnitudes of RAEs among soccer, baseball, and volleyball decreased over time, but those of basketball did not. The exact reasons for these remain unclear, but socio-cultural factors, such as low birthrates and the popularity of sports in Japan, might be related to the changing RAEs.

## Additional File

**Additional file 1.** Supplementary Table 1. The number of the general population (Japanese male) for calculating the age range.

## Data Availability

The data that support the findings of this study are available on request from the final author [H.N.].

## Abbreviations

RAE: Relative age effect
