# Supplementary material for: Relative Age Effects in Male Japanese Professional Athletes: a 25-Year Historical Analysis
Source: Sports Med Open. 2020 Oct 6;6:48. doi: 10.1186/s40798-020-00277-4 (PMC7538493; doi:10.1186/s40798-020-00277-4)
Supplement: Supplementary file 1 — Additional file 1. Supplementary Table 1. The number of the general population (Japanese male) for calculating the age range. [file 40798_2020_277_MOESM1_ESM.docx]

**Supplementary Table 1:** The number of the general population (Japanese male) for calculating the age range

| Registered | Q1 | Q2 | Q3 | Q4 | Total |
| --- | --- | --- | --- | --- | --- |
| 1993 | 3,014,838 | 3,130,750 | 3,000,086 | 3,223,846 | 12,369,520 |
| (1962-1974) | 24.4 (%) | 25.3 (%) | 24.3 (%) | 26.1 (%) |  |
|  |  |  |  |  |  |
| 2001 | 3,025,543 | 3,171,302 | 2,942,809 | 2,929,124 | 12,068,778 |
| (1970-1982) | 25.1 (%) | 26.3 (%) | 24.4 (%) | 24.3 (%) |  |
|  |  |  |  |  |  |
| 2010 | 2,352,425 | 2,493,042 | 2,337,579 | 2,288,824 | 9,471,870 |
| (1979-1991) | 24.8 (%) | 26.3 (%) | 24.7 (%) | 24.2 (%) |  |
|  |  |  |  |  |  |
| 2018 | 2,051,303 | 2,145,750 | 2,025,457 | 1,954,171 | 8,176,681 |
| (1987-1999) | 25.1 (%) | 26.2 (%) | 24.8 (%) | 23.9 (%) |  |
